# Perceptions of Stress and Mood Associated With Listening to Music in Daily Life During the COVID-19 Lockdown

**DOI:** 10.1001/jamanetworkopen.2022.50382

**Published:** 2023-01-10

**Authors:** Anja C. Feneberg, Ana Stijovic, Paul A. G. Forbes, Claus Lamm, Giulio Piperno, Ekaterina Pronizius, Giorgia Silani, Urs M. Nater

**Affiliations:** 1 Department of Clinical and Health Psychology, Faculty of Psychology, University of Vienna, Vienna, Austria; 2 University Research Platform “The Stress of Life (SOLE)—Processes and Mechanisms Underlying Everyday Life Stress,” University of Vienna, Vienna, Austria; 3 Department of Cognition, Emotion, and Methods in Psychology, Faculty of Psychology, University of Vienna, Vienna, Austria

## Abstract

**Importance:**

Music listening is a universal human experience. People of all ages and cultures often use music to reduce stress and improve mood, particularly in times of crisis. However, ecologically valid research examining the real-time association of music listening with stress and mood during the COVID-19 pandemic is scarce.

**Objective:**

To explore the associations between listening to music and the perceptions of stress and mood using ecological momentary assessment during the COVID-19 lockdown period.

**Design, Setting, and Participants:**

In this cohort study conducted between April 1 and May 8, 2020, adults from the general population residing in Austria and Italy were prompted by an app on their smartphone to report data 5 times per day across 7 consecutive days. Participants provided data on their real-time and real-life experiences in their natural environment while strict lockdown measures were in place. Data analysis was performed from March 2021 to February 2022.

**Exposures:**

Data on self-reported music listening were recorded by means of mobile-based assessments. Perceived chronic stress was assessed once at the end of the study.

**Main Outcomes and Measures:**

Perceptions of momentary stress and mood were measured using visual analog scales (score range, 0-100, where 0 indicates not at all and 100 indicates very much) by means of mobile app–based assessments.

**Results:**

The final sample comprised 711 participants (497 women [69.9%]; median age, 27.0 years [IQR, 24.0-36.0 years]). Participants provided a total of 19 641 data points, including 4677 music listening reports. Music listening was prospectively associated with lower momentary stress levels (β, −0.92; 95% CI, −1.80 to −0.04; *P* = .04) and improvements in mood valence (β, 1.90; 95% CI, 1.17-2.63; *P* < .001), especially if the music was perceived as happy. Individuals with higher levels of chronic stress reported improved mood valence after music listening (β, 0.12; 95% CI, 0.02-0.22; *P* = .02).

**Conclusions and Relevance:**

The present findings suggest that music listening may be a means to modulate stress and mood during psychologically demanding periods. Individuals experiencing heightened momentary and/or chronic stress because of the challenges brought about by COVID-19 pandemic–related restrictions might consider music as an easily accessible tool for the management of stress and mood in daily life.

## Key Points

**Question:**

Was listening to music in daily life during COVID-19 pandemic restrictions associated with lower stress and better mood?

**Findings:**

In this cohort study of 711 adults, listening to music was significantly associated with lower levels of stress. Moreover, music listening was significantly associated with improved mood, particularly for those with elevated chronic stress during the COVID-19 pandemic.

**Meaning:**

These findings suggest that listening to music in daily life, a simple intervention, may support beneficial health outcomes during COVID-19 pandemic restrictions.

## Teaser

This cohort study explores the associations between listening to music and the perceptions of stress and mood using ecological momentary assessment during the COVID-19 lockdown period in April and May 2020.

## Introduction

The COVID-19 pandemic has changed the everyday life of many individuals worldwide. Starting in the spring of 2020, many countries implemented far-reaching lockdown measures, including strict social distancing and stay-at-home orders.^1^ The resulting social and economic disruption was associated with stress, worries, and mental health problems among the general population.^2,3,4^ This disruption was aggravated by the fact that many leisure activities that were previously pursued to regulate stress and mood (eg, meeting friends, attending cultural events) were banned because of the lockdown. Prolonged levels of heightened stress (ie, chronic stress) and impaired mood have been shown to be major risk factors for mental and somatic disorders,^5^ specifically in the context of the COVID-19 pandemic.^6,7,8^

Music listening qualifies as an easily accessible coping strategy during times of a pandemic. Music has the capacity to modulate cognitive, affective, and neurobiological processes.^9,10,11,12^ Moreover, historical evidence suggests that, particularly in times of crisis and disasters, individuals across cultures turn to music to lift their mood and to increase feelings of social connectedness.^13^ This evidence is supported by recent research that investigated the role of music during the COVID-19 pandemic.^14^ According to a multinational representative survey, individuals across countries reported an increase in hours spent listening to music during the first COVID-19 lockdown, particularly for the purpose of mood regulation.^15^ Moreover, music listening was found to be associated with lower depressive symptoms and psychological distress during lockdown^16^ and higher life satisfaction, even when compared with other leisure activities, such as watching movies.^17^

A limitation of previous studies in this field is that most were cross-sectional and/or applied retrospective instruments, asking individuals to estimate how they felt and behaved overall across the past week(s) or month. They are thus prone to recall bias and not suited to address the prospective associations between music-listening behavior and health outcomes as they unfolded in daily life during the COVID-19 lockdown period. In contrast, ecological momentary assessment (EMA) involves repeated sampling of participants’ behaviors and experiences in (quasi) real time in their natural environment. Therefore, EMA aims to minimize recall bias and maximize ecological validity and enables the examination of dynamic microprocesses as they occur in real life.^18^ Ecological momentary assessment studies conducted prior to the COVID-19 pandemic have revealed a complex interplay between characteristics of the listener, the situation, and the music in terms of stress and mood regulation.^19,20,21^ In this regard, previous research suggests that the stress-reducing effects of music listening might be limited when individuals are confronted with intense and/or chronic stressors.^19,22,23^ This possibility challenges the assumption of the beneficial effects of music in the context of a global stressor, such as a pandemic.

Taken together, increased levels of stress and impaired mood during the COVID-19 lockdown constitute risk factors for detrimental health outcomes. Music listening qualifies as a helpful means of self-regulation during such times of crisis, as indicated by previous research.^14,15,16,17^ However, the real-time and real-life stress- and mood-regulatory capacities of music listening in daily life remain to be investigated, to our knowledge.

As part of a preregistered research project,^24^ we conducted an EMA study with more than 700 individuals in Austria and Italy when strict lockdown measures were first implemented in both countries (eAppendix in Supplement 1). We hypothesized that music listening would be associated with lower levels of momentary stress and higher levels of mood (valence, energetic arousal, and calmness). We investigated time-delayed prospective (as opposed to concurrent) associations between music listening and momentary stress and mood. To unravel the regulatory properties of music listening, we examined whether the association between music listening and subsequent stress and mood levels varied depending on levels of stress and mood from the last data entry. Furthermore, we examined whether perceived chronic stress moderated the associations between music listening and momentary stress and mood and expected to find benefits associated with music listening for individuals experiencing lower levels of chronic stress.^19^

## Methods

### Participants

To be eligible for inclusion in this cohort study, participants had to be fluent in German or Italian, aged 18 years or older, and own an Android smartphone or tablet. Participants were reimbursed with €20 (US $20.73) and were given the opportunity to take part in a raffle to win €100 (US $103.67). All study procedures were conducted in accordance with the World Medical Association Declaration of Helsinki^25^ and its later amendments. All participants provided electronic consent at the beginning of the study and were informed that they could discontinue study participation at any time. The local ethics review board of the University of Vienna approved the study. This report followed the Strengthening the Reporting of Observational Studies in Epidemiology (STROBE) reporting guideline for observational studies^26^ and the adapted STROBE checklist for reporting EMA studies (CREMAS).^27^

### Procedure

Data collection took place from April 1 to May 8, 2020. Recruitment was undertaken via advertisements in online media outlets and social media (eg, Facebook groups, Twitter), word of mouth, and via an existing study database comprising individuals from the general population. Interested participants e-mailed the study team and received a link to download the app movisensXS (Movisens GmbH) for data collection and a study manual with information regarding the sampling protocol, meaning of items, and contact details in case of questions or technical difficulties. At the beginning of the study, participants entered demographic information into the app and became accustomed to the EMA protocol. The EMA period started the next day and lasted for 7 consecutive days, with 5 data entries scheduled throughout each day. Individuals were prompted by an auditory signal to answer questions randomly between 10:00 to 11:00, 11:00 to 14:00, 14:00 to 17:00, and 17:00 to 20:00, with at least 89 minutes between 2 subsequent prompts and the possibility to postpone a data entry for a maximum delay time of 30 minutes. The fifth data entry of the day had to be self-initiated at bedtime, for which participants received a reminder prompt at 21:00. At the end of the EMA period, participants completed a final online survey (Sosci Survey GmbH). Data analysis was performed from March 2021 to February 2022.

### Ecological Momentary Assessment

Momentary stress was assessed using the item, “At the moment, I feel stressed,” answered on a visual analogue scale ranging from 0 (not at all) to 100 (very much) at each data entry.^28^ Momentary mood was measured based on a validated mood scale with 6 items assessing the 3 basic mood dimensions: valence (unwell-well; discontented-contented), energetic arousal (tired-awake; without energy-full of energy), and calmness (restless-calm; tense-relaxed).^29^ Participants indicated their momentary mood at each data entry using a visual analogue scale ranging from 0 to 100 per item. Higher values indicate higher valence, energetic arousal, or calmness, respectively.

Music listening was assessed in line with previous recommendations for EMA studies.^30^ At each data entry, participants reported whether they were deliberately listening to music at the moment of responding (yes = 1 and no = 0). If they responded with no, participants were asked whether they had been listening to music since the last data entry (or since waking up for the first prompt of the day) (yes = 1 and no = 0). When participants indicated current or past music listening, they were asked to state the perceived music characteristics on a bipolar visual analogue scale (valence: 0 [sad] to 100 [happy]; arousal: 0 [calming] to 100 [energizing]) and to indicate the main reason for music listening (relaxation, activation, distraction, reducing boredom, and no reason; selected = 1 and not selected = 0). Only 1 reason could be selected per response (eMethods 1 in Supplement 1).

### Final (Non-EMA) Online Survey

The 10-item version of the Perceived Stress Scale was administered once at the end of the EMA period to measure chronic stress during the past 4 weeks.^31,32^ The total score ranges from 0 to 40, with higher scores indicating higher chronic stress levels (Cronbach α = 0.88 in this study). Information on depressive symptoms was assessed using a subscale of the Patient Health Questionnaire and served as a covariate in the present study.^33^ To gain an insight into participants’ habitual music-listening behavior, we used a short version of the Music Preference Questionnaire.^34^

### Statistical Analysis

We computed linear multilevel models using RStudio, version 4.2.1 (R Group for Statistical Computing)^35^ with the packages lme4^36^ and interactions.^37^ We computed separate 3-level models (observations nested within days nested within persons) for momentary stress, mood valence, and calmness as dependent variables. For energetic arousal, we computed a 2-level model (observations nested within persons) because the variance on the day level was negligible (eTable 1 in Supplement 1).

The multilevel modeling proceeded in a stepwise manner^38,39^: empty random-intercept model, control model with all covariates including the lagged value of the dependent variable from the last measurement time point (ie, the lag-1 serial autocorrelation),^40^ conditional model including all covariates and the focal variables, conditional model including all covariates, focal variables, and a lower-level interaction between the focal variables and the lagged dependent variable. This enabled us to test whether the association between music listening and momentary stress and mood varied depending on previous levels of stress and mood. For models examining previous music listening, we additionally included the cross-level interaction between perceived chronic stress (person level) and previous music listening (observation level) in a final step. Model comparisons were undertaken by means of χ^2^ statistics, which compare the reduction in deviance as a measure of model fit.

A first set of models examined previous music listening as the focal variable. A second and third set of models examined perceived music characteristics (entered together) and reasons for music listening (entered together) as focal variables, respectively. We ensured that music listening temporarily preceded the dependent variable by lagging reports of current music listening and combining them with reports of music listening since the last data entry or waking up into a single binary variable (0 = no previous music listening; 1 = previous music listening). Data on perceived music characteristics and reasons for music listening were treated accordingly. The number of observations was reduced to reports of music listening in models examining music characteristics and reasons for music listening on the observation level. We additionally included the person means of the focal variables in all models. Thereby, we examined associations between music listening and stress and mood both across time and across individuals.

Details regarding the included covariates, applied centering methods, inclusion of random slopes, and number of observations included in the models are reported in eMethods 2 in Supplement 1. Significant interactions were probed using the Johnson-Neyman test, which provides regions of significance at all levels of the moderator.^41^ We used maximum likelihood for model estimation with listwise deletion in case of missing values. We report unstandardized β coefficients, which indicate the change score in the outcome by a 1-unit increase in the independent variable. The Satterthwaite method was used to test significance using *lmerTest*.^42^ All *P* values were from 2-sided tests and results were deemed statistically significant at *P* < .05. As a measure of effect size, we report pseudo-*R*^2^, representing the change in residual variance or change in intercept variance with (σ^2^ reference model − σ^2^ final model)/σ^2^ reference model.^43^ Data analysis was performed from March 2021 to February 2022.

## Results

The final sample comprised 711 participants (497 women [69.9%]; 68.1% residing in Austria; and median age, 27.0 years [IQR, 24.0-36.0 years]) (eMethods 3 in Supplement 1). Table 1 displays the participants’ characteristics and the means of the EMA reports.^2,20,21,22,39,44^ From a total of 24 885 possible data points (5 × 7 × 711), 5244 (21.1%) did not receive a response, resulting in an overall compliance rate of 78.9% (n = 19 641). Participants reported (current or previous) music listening on 23.8% (n = 4677) of the observations (see eResults 1 in Supplement 1 for details on missing data and study compliance, eResults 2 and 3 in Supplement 1 for details on habitual and daily life music [listening] behavior, and eTable 1 in Supplement 1 for descriptive statistics for dependent variables).

**Table 1.** Study Sample Characteristics and Ecological Momentary Assessment Data^a^

Characteristic	No. (%)		
	Overall (N = 711)	Austria (n = 484)	Italy (n = 227)
Gender			
Female	497 (69.9)	364 (75.2)	133 (58.6)
Male	214 (30.1)	120 (24.8)	94 (41.4)
Age, y			
Median (IQR)	27.0 (24.0-36.0)	29.0 (24.0-40.0)	26.0 (24.0-29.0)
Sample range	18-80	18-76	18-80
Educational level^b^			
Low	33 (4.6)	30 (6.2)	3 (1.3)
Medium	259 (36.4)	190 (39.3)	69 (30.4)
High	419 (58.9)	264 (54.5)	155 (68.3)
Current working status^c^			
Full or part time at home	223 (28.4)	175 (32.1)	48 (20.1)
Full or part time away from home	117 (14.9)	85 (15.6)	32 (13.4)
Full-time student	264 (33.7)	162 (29.7)	102 (42.7)
Unemployed	66 (8.4)	35 (6.4)	31 (13.0)
Other	114 (14.5)	88 (16.1)	26 (10.9)
Perceived chronic stress, median (IQR)^d^	19.0 (13.0-24.0)	17.0 (12.0-24.0)	21.0 (16.0-26.0)
Depressive symptoms, median (IQR)^e^	7.0 (4.0-11.0)	7.0 (4.0-11.0)	8.0 (6.0-12.0)
Ecological momentary assessment			
Momentary stress, median (IQR)^f^	28.3 (15.9-43.0)	24.1 (13.8-35.6)	39.5 (25.6-52.5)
Mood valence, mean (SD)^f^	63.3 (14.5)	65.8 (13.8)	58.1 (14.4)
Energetic arousal, mean (SD)^f^	50.7 (10.7)	51.2 (10.1)	49.6 (11.8)
Calmness, mean (SD)^f^	61.7 (15.4)	64.3 (14.2)	56.1 (16.4)
Music-listening reports	4677 (23.8)	3406 (25.2)	1271 (21.2)
Range per person	0-32	0-32	0-21

^a^ Normality of data was examined using Kolmogorov-Smirnov tests.

^b^ Based on the International Standard Classification of Education 2011^44^ (low, level 1-2; medium, level 3-4; and high, level ≥5).

^c^ Multiple answers were possible.

^d^ Assessed via the 10-item Perceived Stress Scale^20,21^; scale ranges from 0 to 40. Prepandemic normal mean (SD) values were based on a representative German sample^21^: 13 (6.5); mean (SD) values derived from a representative Austrian sample in April 2020^2^: 16.0 (7.5).

^e^ Assessed via the Patient Health Questionnaire^22^; scale ranges from 0 to 27; values of 5 or more indicate mild levels of depression severity and values of 10 or more indicate moderate levels of depression severity. Prepandemic normal mean (SD) values were based on a representative German sample^39^: 2.9 (3.5); mean (SD) values derived from a representative Austrian sample in April 2020^2^: 6.2 (5.4).

^f^ Assessed on visual analogue scales (range, 0-100); displayed values are based on person means.

### Momentary Stress

**Figure 1.**
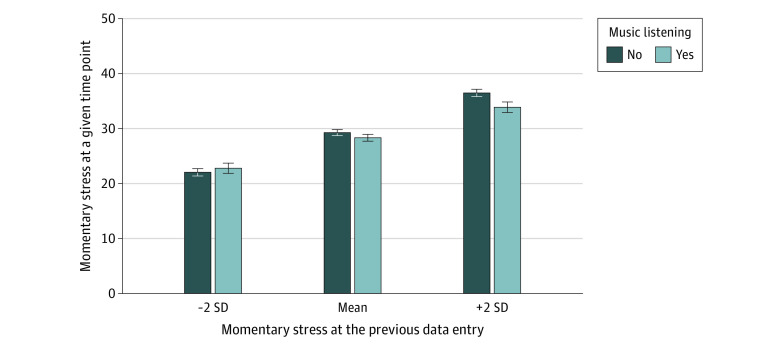
Differential Associations Between Music Listening and Momentary Stress Depending on Prior Stress Levels Momentary stress varied as a function of music listening and prior stress levels. The y-axis displays momentary stress assessed at a given time point. The x-axis displays categories of momentary stress levels at the previous data entry. Error bars represent SEs.

Music listening was prospectively associated with lower momentary stress (β, −0.92; 95% CI, −1.80 to −0.04; *P* = .04) and explained 0.1% of the residual variance (χ^2^_6_ = 5.94; *P* = .43) (eTable 2 in Supplement 1). A significant previous music listening × previous stress level interaction (β, −0.07; 95% CI, −0.11 to −0.02; *P* = .005) indicated that the association between previous music listening and momentary stress varied as a function of the previous stress level and explained no additional residual and 7.1% intercept variance on the day level (χ^2^_1_ = 7.65; *P* = .006) (eTable 3 in Supplement 1). The Johnson-Neyman test indicated that music listening in moments of above-average levels of stress (>0.04 SD) was associated with a significant decrease in stress levels, while music listening in moments of low levels of stress (less than –3.97 SD) was associated with a significant increase in subsequent stress levels (Figure 1). On the person level, music listening was not significantly associated with stress levels; that is, stress levels did not differ between individuals regardless of how frequently they listened to music.

### Mood

**Figure 2.**
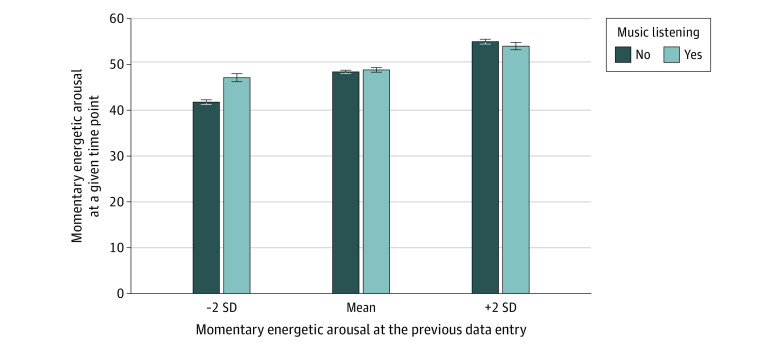
Differential Associations Between Music Listening and Energetic Arousal Depending on Prior Energetic Arousal Levels Energetic arousal varied as a function of music listening and prior energetic arousal levels. The y-axis displays energetic arousal assessed at a given time point. The x-axis displays categories of energetic arousal levels at the previous data entry. Error bars represent SEs.

Music listening was prospectively associated with higher mood valence (β, 1.90; 95% CI, 1.17-2.63; *P* < .001) and calmness (β, 1.39; 95% CI, 0.60-2.17; *P* = .001), irrespective of previous levels of valence and calmness, respectively (eTable 2 in Supplement 1). Music listening explained 0.4% (χ^2^_5_ = 30.08; *P* < .001) of the residual variance in mood valence and 0.3% (χ^2^_6_ = 20.81; *P* = .002) of the residual variance in calmness. Furthermore, music listening was associated with higher energetic arousal (β, 2.04; 95% CI, 1.19-2.89; *P* < .001) and explained 0.7% of the residual variance (χ^2^_6_ = 33.83; *P* < .001). A significant previous music listening × previous energetic arousal interaction (β, −0.09; 95% CI, −0.13 to −0.04; *P* < .001) (eTable 3 in Supplement 1) indicated that this association varied as a function of previous energetic arousal and explained 0.1% of the residual variance (χ^2^_1_ = 12.75; *P* < .001). The Johnson-Neyman test revealed that music listening in moments with energetic arousal levels that were below 0.69 SD was associated with increased subsequent energetic arousal levels, while music listening at higher levels of energetic arousal (≥0.69 SD) was not significantly associated with subsequent energetic arousal (Figure 2). On the person level, music listening was not significantly associated with mood.

### Chronic Stress as a Moderator

Chronic stress moderated the association between music listening and mood valence (β, 0.12; 95% CI, 0.02-0.22; *P* = .02) (eTable 4 in Supplement 1). A comparable result was found for energetic arousal (β, 0.15; 95% CI, 0.03-0.27; *P* = .01). Thus, individuals reporting higher chronic stress showed higher mood valence and energetic arousal after music listening compared with others. Models including the cross-level interaction (previous music listening × chronic stress) explained 0.4% of the residual variance in valence (χ^2^_7_ = 37.61; *P* < .001) and 0.8% of the residual variance in energetic arousal (χ^2^_8_ = 52.33; *P* < .001) compared with the control model. Johnson-Neyman tests revealed that music listening was associated with improved mood valence starting at Perceived Stress Scale values greater than 12.1 and with energetic arousal starting at Perceived Stress Scale values greater than 10.9. The associations between previous music listening and momentary stress and calmness were not moderated by chronic stress.

### Role of Music Characteristics and Reasons for Music Listening

A summary of the main results is shown in Table 2. On the observation level, listening to happier music (relative to a person’s average reports) was significantly associated with lower momentary stress and improvements in mood valence and calmness. A significant music valence × previous mood valence interaction indicated that listening to happier music at levels of mood valence that were below 0.43 SD was associated with an increase in subsequent mood valence (β, −0.00; 95% CI, −0.01 to −0.00; *P* < .001; with 1.6% explained residual variance, χ^2^_1_ = 13.66; *P* < .001). Moreover, listening to more energizing music (relative to a person’s average reports) was significantly associated with higher energetic arousal. On the person level, the results showed that individuals who listened to happier music reported lower levels of stress and improved mood on all dimensions compared with others. Except for energetic arousal, the same pattern of person-level differences emerged when examining associations across all available data points.

**Table 2.** Associations Between Music Characteristics and Reasons for Music Listening and Dependent Variables Across Time (Observation Level) and Across Individuals (Person Level)^a^

Independent variable	Momentary stress			Mood valence			Energetic arousal			Calmness		
	β (95% CI)	*P* value	Pseudo-*R*^2^, %	β (95% CI)	*P* value	Pseudo-*R*^2^, %	β (95% CI)	*P* value	Pseudo-*R*^2^, %	β (95% CI)	*P* value	Pseudo-*R*^2^, %
**Observation level**												
Musical valence	−0.07 (−0.12 to −0.02)	.003^b^	<.001	0.08 (0.04 to 0.12)	<.001^b^	<.001	0.04 (−0.01 to 0.09)	.11	NA	0.08 (0.04 to 0.12)	<.001^b^	0.08
Musical arousal	0.01 (−0.03 to 0.04)	.72	NA	0.01 (−0.02 to 0.04)	.61	NA	0.06 (0.02 to 0.09)	.002^b^	0.50	−0.00 (−0.04 to 0.03)	.82	NA
Relaxation	1.91 (−0.93 to 4.74)	.19	NA	0.08 (−2.33 to 2.49)	.95	NA	−0.87 (−3.59 to 1.86)	.53	NA	−0.50 (−3.05 to 2.04)	.70	NA
Activation	1.34 (−1.46 to 4.14)	.35	NA	0.82 (−1.56 to 3.21)	.50	NA	1.23 (−1.47 to 3.94)	.37	NA	0.76 (−1.76 to 3.27)	.56	NA
Distraction	4.16 (1.04 to 7.29)	.009^b^	1.04	−1.82 (−4.48 to 0.84)	.18	NA	0.90 (−2.11 to 3.91)	.56	NA	−1.36 (−4.16 to 1.45)	.34	NA
Reducing boredom	1.21 (−2.14 to 4.57)	.48	NA	0.17 (−2.69 to 3.03)	.91	NA	1.39 (−1.85 to 4.62)	.40	NA	0.70 (−2.32 to 3.72)	.65	NA
**Person level** ^c^												
Musical valence	−0.17 (−0.27 to −0.08)	<.001^b^	3.88	0.20 (0.12 to 0.28)	<.001^b^	6.90	0.11 (0.04 to 0.19)	.004^b^	4.75	0.18 (0.10 to 0.27)	<.001^b^	5.04
Musical arousal	−0.02 (−0.10 to 0.07)	.72	NA	0.01 (−0.06 to 0.09)	.71	NA	0.03 (−0.04 to 0.10)	.47	NA	0.01 (−0.07 to 0.09)	.85	NA
Relaxation	−1.10 (−7.00 to 4.80)	.72	NA	5.73 (0.64 to 10.81)	.03^b^	1.21	−1.71 (−6.42 to 2.99)	.48	NA	8.54 (3.28 to 13.80)	.001^b^	2.48
Activation	−4.09 (−9.66 to 1.47)	.15	NA	7.56 (2.77 to 12.35)	.002^b^	2.60	4.53 (0.08 to 8.98)	.046^b^	1.64	9.27 (4.31 to 14.23)	<.001^b^	2.39
Distraction	−1.93 (−9.01 to 5.14)	.59	NA	1.32 (−4.77 to 7.42)	.67	NA	−2.94 (−8.57 to 2.70)	.31	NA	3.89 (−2.42 to 10.19)	.23	NA
Reducing boredom	0.16 (−7.87 to 8.19)	.97	NA	3.57 (−3.35 to 10.49)	.31	NA	4.83 (−1.54 to 11.19)	.14	NA	6.35 (−0.80 to 13.51)	.08	NA

Abbreviation: NA, not applicable.

^a^ Models are based on 2664 observations (momentary stress, mood valence, and calmness) and 2665 observations (energetic arousal) from 599 individuals.

^b^ Significant at *P* < .05.

^c^ On the observation level, person-mean–centered variables were entered. On the person level, person means (ie, means per person across the study period) of observed values were entered as variables. Pseudo-*R*^2^ indicates the reduction in residual variance (observation level) and reduction in intercept variance (person level), respectively, relative to models excluding the respective variables. Although musical valence did not explain residual variance on the observation level for momentary stress and mood valence, it did explain substantial intercept variance on the day level with 14.6% for momentary stress and 25.2% for mood valence. Displayed are fixed effects. Full models including covariates and random effects are provided in eTables 5 and 6 in Supplement 1.

Regarding reasons for music listening, listening to music for the reason of distraction was prospectively associated with higher momentary stress. Furthermore, a distraction × previous mood valence interaction showed that listening to music for distraction at above-average values of mood valence (>0.57 SD) was associated with a decrease in mood valence (β, −0.19; 95% CI, −0.37 to −0.02; *P* = .03; pseudo-*R*^2^ = 0.5%; χ^2^_1_ = 4.84; *P* = .03). No other reasons were significantly associated with momentary stress or mood. On the person level, individuals who listened to music more frequently for the reasons of relaxation and activation reported higher levels of mood valence, energetic arousal, and calmness compared with others. However, these person-level differences did not emerge when examining associations across all available data points.

## Discussion

In this preregistered EMA cohort study, we examined whether music listening in daily life was associated with perceptions of stress and mood while strict social distancing and stay-at-home orders were in place during the COVID-19 lockdown. More than 700 individuals reported their music-listening behavior and momentary stress and mood levels 5 times per day using a smartphone app. Our findings suggest that music listening was a beneficial tool for stress and mood management during lockdown. Happy music, in particular, was associated with lower stress levels and improved mood across time and across individuals. Furthermore, individuals reporting higher chronic stress levels reported improved mood after music listening.

The present study corroborates and extends previous research that has highlighted the health benefits associated with music and the value of music in coping with psychological distress during the COVID-19 lockdown.^14,15,16,17,45,46^ The present study provides unique evidence from a real-life and real-time perspective on the prospective associations between deliberate music listening and perceptions of stress and mood during lockdown among a large sample of individuals from the general population.

In this regard, our findings indicate that music listening in daily life might regulate levels of stress and energetic arousal toward an optimal state while it improves mood valence and calmness. Thus, individuals might use music to purposefully upregulate or downregulate arousal-related states toward an optimal balance while increasing hedonic valence. This notion is broadly in line with findings from a prepandemic EMA study^21^ and mood management theory more generally.^47^

Contrary to our expectations, individuals reporting higher levels of chronic stress experienced the most benefit associated with music listening in terms of improved mood valence and energetic arousal. Previous studies conducted in the context of a stressful student examination period^19^ and an experimental psychosocial stress paradigm^9^ suggested that the benefits associated with music might be limited under heightened stress. However, these stressful situations may not be comparable with the COVID-19 pandemic. Our findings are in line with previous cross-sectional research conducted during early stages of the pandemic. Fink and colleagues^15^ showed that individuals reporting more negative emotions and distress because of the pandemic engaged with music for the specific purpose of emotional coping and mood regulation. Considering the detrimental mental and physical consequences that can arise from chronic stress in the context of COVID-19,^6^ music listening might be considered an easy intervention.

Finally, we found differential associations between music characteristics and reasons for music listening and stress and mood. On the observation level, individuals reported feeling more awake and energized after listening to more energizing music than was usual for them, while the opposite was reported after listening to more calming music. This finding corresponds well with findings from experimental and daily-life studies that found musical arousal and associated objective music characteristics (eg, tempo) modulated the activity of the autonomic nervous system,^48^ which is responsible for the regulation of psychophysiological arousal. Furthermore, musical valence showed associations with stress and mood both across time and across individuals. On the one hand, listening to happier music was prospectively associated with lower stress and improved mood in daily life. This finding might be explained by neurobiological alterations because emotions conveyed by musical valence (eg, sadness, happiness) elicit activity in the amygdala, the hippocampus, and the ventral striatum, which are important brain regions involved in emotion regulation.^10^ On the other hand, individuals with a preference for happy music displayed lower levels of stress and improved mood than others. A previous study reported that those listening to happy music more during the pandemic compared with before used more adaptive regulation strategies, such as cognitive reappraisal,^46^ which might translate into lower stress and improved mood. In addition, listening to music for distraction was associated with higher stress levels and lower mood valence. Distraction by music might be functional to cope with acute (daily) stressors but might be a maladaptive coping strategy when facing long-term stressors,^19^ such as the COVID-19 lockdown. In line with this idea, distraction was reported as one of the most commonly applied strategies but not the most effective strategy to cope with challenges during the pandemic.^49^

### Limitations

Our findings need to be interpreted in light of several limitations. First, we recruited a convenience sample, which is not representative of the general population. Second, effect sizes tended to be small, although they may nevertheless indicate clinically relevant findings due to the low cost and easy availability of music in daily life. Third, as this study was observational, additional confounders cannot be ruled out. Although we included a comprehensive set of covariates and considered a temporal precedence of up to several hours between reports of music listening and the measurement of stress and mood, these requirements do not suffice to determine causality. Fourth, we assessed only whether music listening had occurred. Thus, we do not know how far back in time it occurred and which music, exactly, participants listened to in terms of genre or objective music characteristics. Fifth, this study lacks a prelockdown and postlockdown control condition. Therefore, future studies will need to determine the extent to which the present findings were specific to lockdown.

## Conclusions

Because music is highly popular across cultures and age groups and readily available at almost no cost, music listening can be considered a low-threshold intervention to improve health and well-being on the population level during times of crisis. The present study improves our understanding of stress and mood management using music during COVID-19–related lockdowns and revealed that individuals experiencing heightened momentary and/or chronic stress may experience the most benefit associated with music listening. Our findings can further promote the development of individualized and tailored interventions that deliver music to foster resilience in daily life during psychologically demanding periods.

## Notes

Supplement 1. **eAppendix.** Study Background and Preregistered Hypotheses **eMethods 1.** Details on the Assessment of Music Listening in Daily Life Participant Attrition and Data Cleaning **eMethods 2.** Details on the Analytical Approach **eMethods 3.** Participant Attrition and Data Cleaning **eResults 1.** Missing Data and Study Compliance **eResults 2.** Details on Habitual Music Listening Behavior **eResults 3.** Details on Music-Listening Behavior in Everyday Life **eTable 1.** Descriptive Statistics for Outcome Variables **eTable 2.** Multilevel Models Predicting Dependent Variables by Music Listening and Covariates **eTable 3.** Multilevel Models Predicting Dependent Variables by Music Listening, the Interaction Between Music Listening and Lagged DV, and Covariates **eTable 4.** Multilevel Models Predicting Dependent Variables by Music Listening, the Interaction Between Music Listening and Lagged DV, the Interaction Between Music Listening and Chronic Stress (PSS), and Covariates **eTable 5.** Multilevel Models Predicting Dependent Variables by Music Characteristics and Covariates **eTable 6.** Multilevel Models Predicting Dependent Variables by Reasons for Music Listening and Covariates
eReferences.

Supplement 2.
Data Sharing Statement
