# Supplementary material for: Perceptions of Stress and Mood Associated With Listening to Music in Daily Life During the COVID-19 Lockdown
Source: JAMA Netw Open. 2023 Jan 10;6(1):e2250382. doi: 10.1001/jamanetworkopen.2022.50382 (PMC9857599; doi:10.1001/jamanetworkopen.2022.50382)
Supplement: Supplement 1. — eAppendix. Study Background and Preregistered Hypotheses eMethods 1. Details on the Assessment of Music Listening in Daily Life Participant Attrition and Data Cleaning eMethods 2. Details on the Analytical Approach eMethods 3. Participant Attrition and Data Cleaning eResults 1. Missing Data and Study Compliance eResults 2. Details on Habitual Music Listening Behavior eResults 3. Details on Music-Listening Behavior in Everyday Life eTable 1. Descriptive Statistics for Outcome Variables eTable 2. Multilevel Models Predicting Dependent Variables by Music Listening and Covariates eTable 3. Multilevel Models Predicting Dependent Variables by Music Listening, the Interaction Between Music Listening and Lagged DV, and Covariates eTable 4. Multilevel Models Predicting Dependent Variables by Music Listening, the Interaction Between Music Listening and Lagged DV, the Interaction Between Music Listening and Chronic Stress (PSS), and Covariates eTable 5. Multilevel Models Predicting Dependent Variables by Music Characteristics and Covariates eTable 6. Multilevel Models Predicting Dependent Variables by Reasons for Music Listening and Covariates eReferences. [file jamanetwopen-e2250382-s001.pdf]

Supplementary Online Content

Feneberg AC, Stijovic A, Forbes PAG, et al. Perceptions of stress and mood associated with listening to music in daily life during the COVID-19 lockdown. *JAMA Netw Open.* 2023;6(1):e2250382. doi:10.1001/jamanetworkopen.2022.50382

eAppendix. Study Background and Preregistered Hypotheses
eMethods 1. Details on the Assessment of Music Listening in Daily Life Participant Attrition and Data Cleaning
eMethods 2. Details on the Analytical Approach
eMethods 3. Participant Attrition and Data Cleaning
eResults 1. Missing Data and Study Compliance
eResults 2. Details on Habitual Music Listening Behavior
eResults 3. Details on Music-Listening Behavior in Everyday Life
eTable 1. Descriptive Statistics for Outcome Variables
eTable 2. Multilevel Models Predicting Dependent Variables by Music Listening and Covariates
eTable 3. Multilevel Models Predicting Dependent Variables by Music Listening, the Interaction Between Music Listening and Lagged DV, and Covariates
eTable 4. Multilevel Models Predicting Dependent Variables by Music Listening, the Interaction Between Music Listening and Lagged DV, the Interaction Between Music Listening and Chronic Stress (PSS), and Covariates
eTable 5. Multilevel Models Predicting Dependent Variables by Music Characteristics and Covariates
eTable 6. Multilevel Models Predicting Dependent Variables by Reasons for Music Listening and Covariates
eReferences.

This supplementary material has been provided by the authors to give readers additional information about their work.

© 2023 Feneberg AC et al. *JAMA Network Open.*

eAppendix. Study Background and Preregistered Hypotheses

The present study is part of a larger research project entitled "The COVID-19 crisis, stress, mood, and behavior: an ecological momentary assessment study" with the overall aim to examine risk and resilience factors during and after the COVID-19 pandemic from a daily life perspective (see https://osf.io/gsvdf for the preregistered methods and hypotheses).

Strict lockdown measures were implemented in both countries in which we recruited participants, i.e., Austria and Italy, during the data collection period (April 1st to May 8th 2020). See https://osf.io/374qn/ for an overview on restrictions in place during data collection.

At the time of preregistration, we planned to conduct another wave of measurement under 'normal' conditions for comparison purposes (i.e., lockdown vs. 'normal'). However, the pandemic was still ongoing at the date of writing and unpredictability with regard to the future development of the pandemic characterized the majority of the year of 2021 in Austria and Italy. Therefore, we decided to analyze the data from the first lockdown and publish the research results in order to make it available to the scientific community. Consequently, in the present study, we could investigate only part of the preregistered hypotheses. Hypothesis testing can be considered confirmatory in these cases; whilst our examination regarding the moderating role of chronic stress is considered exploratory in nature.

See also Feneberg et al. (2022),[1] Forbes et al. (2022),[2] and Stijovic et al. (2022)[3] for further articles related to the project.

© 2023 Feneberg AC et al. *JAMA Network Open.*

eMethods 1. Details on the Assessment of Music Listening in Daily Life Participant Attrition and Data Cleaning

Deliberate or active music listening was defined as situations in which music listening was the main activity that participants were engaged in, or an accompanying activity with music being in the focus of attention. This excluded background music which was not listened to actively as well as music making (e.g., playing an instrument). This definition was provided in the study manual and could be accessed through the app during the whole study period.

© 2023 Feneberg AC et al. *JAMA Network Open.*

eMethods 2. Details on the Analytical Approach

In all multilevel models, we controlled for a set of covariates. Selection of covariates was based on theoretical and empirical considerations. On the observation level, *time of day* (in hours, centered on 10:00) and *current activity* (work/study/other=0 vs. leisure=1) and on the day level, *weekday* (weekday=0 vs. weekend/holiday=1), were included as covariates. Furthermore, a *quadratic time trend* was added on the observation level to models examining momentary stress and energetic arousal, as this significantly improved model fit. On the person level, *country of residence* (Austria=0, Italy=1), *gender* (woman=0, man=1), *age*, and *depressive symptoms* were included as covariates.

Continuous person-level variables were grand-mean centered (by subtracting the overall sample mean from the individual's value), while binary variables remained uncentered in order to facilitate interpretation of the results. Observation level variables were person-mean centered (by subtracting the person's mean from the value of a given data entry) in order to disentangle within- from between-person effects.[7] *Time of day*, the *quadratic time trend* and *weekday* remained uncentered.

We added random slopes for the focal variable(s) if this did not result in convergence issues.[8] Furthermore, random slopes were added for *time of day* and *current activity* if this did not result in convergence issues and improved model fit.[9]

Lagging of variables is typically related to loss of data. In the present analyses, the lagged value of the dependent variable as well as lagged versions of current music listening were created. Furthermore, 67 out of the 711 participants did not report any music listening episode. Data from these individuals is included in analyses investigating the associations between music listening per se and momentary stress/mood but cannot be included in analyses including music characteristics and reasons for music listening as variables, as analyses with these focal variables are based on reports of music listening. Missing values were excluded listwise by the statistical program. For these reasons, the final number of observations included in the multilevel models ranged from n=2,664 to n=11,987.

eMethods 3. Participant Attrition and Data Cleaning

A total of 2,014 links (for coupling the EMA app and start participation in the study) were sent to individuals that contacted the research team via email and showed initial interest in the study. Of those, 1,211 downloaded the app, provided informed consent to participate, and started at least one data entry. During data cleaning, 260 subjects needed to be excluded since they did not provide enough data (e.g., due to technical problems or loss of interest), so that data from 951 individuals remained with at least 18 (out of 35) observations (including missings).

Of these 951 participants, 198 individuals were excluded (n=2 did not provide sociodemographic information, n=19 were under the age of 18 years, n=57 reported residing in countries other than Austria or Italy, n=119 did not provide data on chronic stress, and n=1 reported being of diverse gender and needed to be excluded from the present analyses as these include gender as covariate). Moreover, current guidelines emphasize the need to consider thresholds of compliance for multilevel analyses. A low compliance rate can be associated with a systematic bias in the data and estimates for lagged effects tend to be less reliable.[4] Based on an a priori power simulation (see https://osf.io/y5atr/ for details), we decided for a 50% completion rate, which has been applied in other studies (see for example Doerr et al., 2015; Mewes et al., 2021).[5,6] Fourty-two individuals were excluded following this criterion. Consequently, 711 participants remained as the final sample in the present study.

© 2023 Feneberg AC et al. *JAMA Network Open.*

eResults 1. Missing Data and Study Compliance

In the final sample (N=711), a total of 24,885 observations were expected according to the EMA protocol (5 observations*7 days*711 subjects). Of these, 5,244 (21.1%) were missing (i.e., signal not responded to/bedtime data entry not initiated), resulting in an overall satisfactory compliance rate of 78.9%. Thus, a participant provided on average 28 data entries (range: 18 – 35 per participant). Furthermore, we discarded 70 (0.3%) data entries due to implausibility indicating potential incompliance with the study protocol (bedtime entry after 6:00 o'clock the next day, response 1 hour after the prompt signal, response duration ≥ 20 min), increasing the number of missing data to a total of 5,313 (21.4%). On 86 (0.3%) observations, data entries were incomplete. Response latency (i.e., amount of time elapsed from prompt signal to answering the prompt) averaged 2 min 56 sec (± 6 min 27 sec). Response duration (i.e., amount of time elapsed from starting to finishing the answer) averaged 2 min 40 sec (± 1 min 36 sec).

To test whether compliance was related to demographic aspects or other study variables, we conducted a multilevel logistic regression analysis in which *missing occurrence* (1=data entry missing, 0=data entry not missing) was modelled as outcome variable. Earlier *measurement time point* during the day (OR = 0.95, p<.001), and later *study day* during the EMA period (OR = 1.13, p<.001) were significantly associated with a higher probability of missing occurrence, indicating lower compliance earlier the day and as the study progressed. Moreover, younger individuals (OR = 0.99, p<.001) and individuals residing in Italy (OR = 1.23, p=.002) showed a higher probability of missing occurrence. In contrast, *day of week* (weekend vs. weekday), *gender*, *chronic stress*, and *depressive symptoms* were unrelated to missing occurrence (ps≥0.10). *Time of day*, *age*, and *country of residence* were included as covariates in all statistical analyses. *Study day* was only significantly associated with *energetic arousal* (i.e., individuals seemed to report higher energetic arousal towards the end of the study period). Since inclusion of *study day* did not markedly alter the final result pattern (data not shown), we decided to refrain from including *study day* as a covariate in the analyses for reasons of parsimony.

© 2023 Feneberg AC et al. *JAMA Network Open.*

eResults 2. Details on Habitual Music Listening Behavior

Habitual music listening behavior was assessed at the final online survey via a shortened version of the Music Preference Questionnaire (revised version), with a focus on main reasons and occasions for music listening, current active music making, and importance of music in life.[10] Participants indicated the habitual frequency of listening to music for various reasons and occasions on Likert scales ranging from 1 (‘never’) to 5 (‘very often’) as well as importance of music in life on a Likert scale ranging from 1 (‘not at all important’) to 5 (‘very important’).

Participants rated music as rather important in their life (3.9±1.1; range: 1 – 5). *Activation* (3.7±1.3), *for the music* (3.5±1.4), and *relaxation* (3.3±1.2) were stated the most frequent reasons to engage in music listening, followed by *distraction* (3.1±1.4), *to evoke feelings* (3.0±1.4), *to intensify feelings* (3.0±1.4), *to reduce boredom* (2.7±1.4), *to work better* (2.5±1.4), *to reduce loneliness* (2.7±1.4), and *to reduce aggression* (1.8±1.2). The main occasions for music listening were *as background activity while engaged in another task* (4.0±1.2), *when being alone* (3.4±1.2), and *when being with friends* (2.4±1.2). Moreover, 27.7% (n=197) of the participants indicated being currently musically active (of those, 23.6% reported playing an instrument and 4.2% reported being member of a choir).

© 2023 Feneberg AC et al. *JAMA Network Open.*

eResults 3. Details on Music-Listening Behavior in Everyday Life

Overall, participants reported a total of 4,677 (23.8%) music listening episodes. On 1,713 (8.8%) observations, participants listened to music at the moment of the data assessment and on 2,964 (15.2%) observations, participants reported music listening since the last data entry. Sixty-seven out of the 711 participants did not report any music listening episode. Music listening occurred most frequently in the afternoon/evening: 17.9% of all music episodes occurred in the morning before/at the first measurement time point (10:00-11:00), 19.0% occurred around noon (11:00-14:00), 21.1% occurred in the afternoon (14:00-17:00), 21.3% occurred in the evening (17:00-20:00), and 20.8% occurred before/when going to bed.

Regarding characteristics of the music, perceived *musical valence* was rated rather happy (65.8±23.1) and perceived *musical arousal* was rated rather energizing (61.3±27.6). *Activation* was stated the most common main reason for music listening (31.6%), followed by *relaxation* (26.6%), *other reasons* (16.2%), *distraction* (14.9%), and *reducing boredom* (10.5%). Descriptively, while *activation* was indicated the most common reason for music listening at all signaled time points during the day, *relaxation* was indicated most frequently at bedtime.

© 2023 Feneberg AC et al. *JAMA Network Open.*

eTable 1. Descriptive Statistics for Outcome Variables

Measure	ICC_{ID}	ICC_{Day}	***M***	SD_{within}	$SD_{betweenID}$	$SD_{betweenDay}$	**1**	**2**	**3**	**4**
1. Momentary stress	0.450	0.093	29.91	17.41	17.28	7.84	1	–0.71***	–0.42***	–0.79***
2. Mood valence	0.432	0.126	63.47	14.02	13.87	7.50	–0.47***	1	0.66***	0.91***
3. Energetic arousal	0.186	0.00	50.89	20.77	9.91	-	0.01	0.32***	1	0.58***
4. Calmness	0.446	0.101	61.92	14.95	14.85	7.07	–0.57***	0.70***	0.10***	1

Notes. Intraclass correlation coefficients (ICC), means (M), standard deviations (SD) and correlations regarding perceived stress and mood dimensions. The correlation matrix shows Pearson correlation coefficients within participants (N=711) below the diagonal and between participants above the diagonal. **p*<.05, ***p*<.01, ****p*<.001.

© 2023 Feneberg AC et al. *JAMA Network Open.*

eTable 2. Multilevel Models Predicting Dependent Variables by Music Listening and Covariates

	**Momentary stress**			**Mood valence**			**Energetic arousal**			**Calmness**		
Independent variable	*b*	*95%CI*	*p*	*b*	*95%CI*	*p*	*b*	*95%CI*	*p*	*b*	*95%CI*	*p*
(Intercept)	28.76	26.40 – 31.12	**<0.001**	65.13	63.38 – 66.88	**<0.001**	55.24	53.25 – 57.24	**<0.001**	61.41	59.56 – 63.27	**<0.001**
Music (pmc)	-0.92	-1.80 – -0.04	**0.041**	1.90	1.17 – 2.63	**<0.001**	2.04	1.19 – 2.89	**<0.001**	1.39	0.60 – 2.17	**0.001**
Time	-0.10	-0.42 – 0.22	0.549	-0.11	-0.18 – -0.04	**0.001**	0.81	0.49 – 1.14	**<0.001**	0.20	0.12 – 0.28	**<0.001**
Time²	-0.02	-0.04 – -0.01	**0.011**				-0.21	-0.22 – -0.19	**<0.001**			
Leisure (pmc)	-5.48	-6.42 – -4.54	**<0.001**	3.25	2.50 – 4.00	**<0.001**	0.61	-0.18 – 1.40	0.127	4.04	3.28 – 4.79	**<0.001**
Weekday	-1.63	-2.36 – -0.90	**<0.001**	0.72	0.10 – 1.34	**0.024**	1.00	0.35 – 1.65	**0.003**	1.35	0.71 – 1.99	**<0.001**
Lagged DV (pmc)	0.18	0.17 – 0.20	**<0.001**	0.24	0.23 – 0.26	**<0.001**	0.17	0.15 – 0.19	**<0.001**	0.20	0.18 – 0.22	**<0.001**
Music (PM)	-0.49	-6.33 – 5.34	0.868	3.89	-0.99 – 8.78	0.118	1.72	-2.19 – 5.64	0.388	3.76	-1.35 – 8.86	0.149
PSS (GMC)	1.02	0.82 – 1.22	**<0.001**	-0.74	-0.91 – -0.57	**<0.001**	-0.22	-0.36 – -0.09	**0.001**	-0.93	-1.10 – -0.75	**<0.001**
Gender	1.46	-0.91 – 3.82	0.227	-2.05	-4.02 – -0.07	**0.043**	2.60	1.02 – 4.18	**0.001**	-2.39	-4.45 – -0.32	**0.024**
Age (GMC)	-0.11	-0.20 – -0.01	**0.033**	-0.02	-0.10 – 0.06	0.662	0.02	-0.05 – 0.08	0.582	0.05	-0.03 – 0.14	0.222
PHQ9 (GMC)	0.55	0.28 – 0.83	**<0.001**	-0.80	-1.03 – -0.57	**<0.001**	-0.30	-0.49 – -0.12	**0.001**	-0.59	-0.83 – -0.35	**<0.001**
Country	10.96	8.57 – 13.35	**<0.001**	-4.50	-6.49 – -2.50	**<0.001**	1.28	-0.32 – 2.88	0.117	-4.83	-6.92 – -2.74	**<0.001**
**Random Effects**												
σ^2	285.86			203.85			253.25			223.30		
τ_{00}	9.80 $_{\text{ID:Day}}$			9.81 $_{\text{ID:Day}}$			240.97 $_{\text{ID}}$			7.95 $_{\text{ID:Day}}$		
	224.90 $_{\text{ID}}$			125.68 $_{\text{ID}}$						151.13 $_{\text{ID}}$		
τ_{11}	0.24 $_{\text{ID.Time}}$			23.20 $_{\text{ID. Leisure\_pmc}}$			2.01 $_{\text{ID.Time}}$			0.26 $_{\text{ID.Time}}$		
	45.72 $_{\text{ID. Leisure\_pmc}}$			2.27 $_{\text{ID. Music pmc}}$			15.27 $_{\text{ID. Leisure\_pmc}}$			17.23 $_{\text{ID. Leisure\_pmc}}$		
	4.12 $_{\text{ID. Music pmc}}$						8.73 $_{\text{ID. Music pmc}}$			5.90 $_{\text{ID. Music pmc}}$		
ρ_{01}	-0.47 $_{\text{ID.Time}}$			0.01 $_{\text{ID.Leisure pmc}}$			-0.84			-0.29 $_{\text{ID.Time}}$		
	-0.21 $_{\text{ID.Leisure pmc}}$			0.05 $_{\text{ID.Music pmc}}$			0.28			-0.15 $_{\text{ID.Leisure pmc}}$		
	-0.13 $_{\text{ID.Music pmc}}$						-0.23			-0.25 $_{\text{ID.Music pmc}}$		
ICC	0.42			0.41			0.31			0.41		
N	711 $_{\text{ID}}$			711 $_{\text{ID}}$			711 $_{\text{ID}}$			711 $_{\text{ID}}$		
	7 $_{\text{Day}}$			7 $_{\text{Day}}$						7 $_{\text{Day}}$		
Observations	11981			11985			11987			11984		
Marginal R^2 / Conditional R^2	0.259 / 0.572			0.230 / 0.544			0.297 / 0.514			0.233 / 0.547		

Notes. Country=0(Austria)/1(Italy), DV=dependent variable, Gender=0(woman)/1(man), Leisure=0(no leisure activity)/1(leisure activity), Music=0(no previous music listening)/1(previous music listening), PHQ9=depressive symptom subscale of the Patient Health Questionnaire,[11] PSS=Perceived Stress Scale,[12] Time was centered on 10:00, Weekday=0(weekday)/1(weekend/holiday); GMC=grand mean centered, PM=person mean, pmc=person mean centered. Significant associations (p<.05) are marked in bold.

© 2023 Feneberg AC et al. *JAMA Network Open.*

eTable 3. Multilevel Models Predicting Dependent Variables by Music Listening, the Interaction Between Music Listening and Lagged DV, and Covariates

	**Momentary stress**			**Mood valence**			**Energetic arousal**			**Calmness**		
Independent variable	*b*	*95%CI*	*p*	*b*	*95%CI*	*p*	*b*	*95%CI*	*p*	*b*	*95%CI*	*p*
(Intercept)	28.77	26.41 – 31.13	**<0.001**	65.13	63.38 – 66.88	**<0.001**	55.25	53.26 – 57.24	**<0.001**	61.42	59.56 – 63.27	**<0.001**
Music (pmc)	-0.84	-1.72 – 0.04	0.062	1.92	1.18 – 2.66	**<0.001**	2.60	1.69 – 3.50	**<0.001**	1.37	0.58 – 2.16	**0.001**
Lagged DV (pmc)	0.18	0.17 – 0.20	**<0.001**	0.24	0.23 – 0.26	**<0.001**	0.17	0.15 – 0.19	**<0.001**	0.20	0.18 – 0.22	**<0.001**
Time	-0.10	-0.42 – 0.22	0.539	-0.11	-0.18 – -0.04	**0.001**	0.81	0.49 – 1.14	**<0.001**	0.20	0.12 – 0.28	**<0.001**
Time²	-0.02	-0.04 – -0.01	**0.012**				-0.21	-0.22 – -0.19	**<0.001**			
Leisure (pmc)	-5.50	-6.43 – -4.57	**<0.001**	3.25	2.50 – 4.00	**<0.001**	0.61	-0.18 – 1.40	0.128	4.04	3.28 – 4.79	**<0.001**
Weekday	-1.63	-2.35 – -0.90	**<0.001**	0.71	0.09 – 1.33	**0.025**	1.00	0.35 – 1.65	**0.003**	1.34	0.70 – 1.98	**<0.001**
Music (PM)	-0.57	-6.40 – 5.26	0.847	3.91	-0.97 – 8.79	0.117	1.90	-2.01 – 5.81	0.342	3.77	-1.34 – 8.87	0.148
PSS (GMC)	1.02	0.82 – 1.22	**<0.001**	-0.74	-0.91 – -0.57	**<0.001**	-0.22	-0.36 – -0.09	**0.001**	-0.93	-1.10 – -0.75	**<0.001**
Gender	1.45	-0.91 – 3.81	0.228	-2.05	-4.03 – -0.07	**0.042**	2.58	1.00 – 4.16	**0.001**	-2.39	-4.46 – -0.32	**0.024**
Age (GMC)	-0.11	-0.20 – -0.01	**0.032**	-0.02	-0.10 – 0.06	0.660	0.02	-0.05 – 0.08	0.596	0.05	-0.03 – 0.14	0.223
PHQ9 (GMC)	0.55	0.28 – 0.83	**<0.001**	-0.80	-1.03 – -0.57	**<0.001**	-0.30	-0.48 – -0.11	**0.002**	-0.59	-0.83 – -0.35	**<0.001**
Country	10.94	8.55 – 13.33	**<0.001**	-4.49	-6.49 – -2.49	**<0.001**	1.29	-0.31 – 2.90	0.114	-4.82	-6.91 – -2.73	**<0.001**
Music (pmc) * lagged DV (pmc)	-0.07	-0.11 – -0.02	**0.005**	-0.03	-0.08 – 0.02	0.238	-0.09	-0.13 – -0.04	**<0.001**	-0.03	-0.07 – 0.02	0.266
**Random Effects**												
σ^2	286.26			203.95			252.98			223.43		
τ_{00}	9.10 $_{ID:Day}$			9.64 $_{ID:Day}$			241.17 $_{ID}$			7.73 $_{ID:Day}$		
	224.45 $_{ID}$			125.69 $_{ID}$						151.14 $_{ID}$		
τ_{11}	0.24 $_{ID.Time}$			23.13 $_{ID. Leisure\_pmc}$			2.00 $_{ID.Time}$			0.26 $_{ID.Time}$		
	45.00 $_{ID. Leisure\_pmc}$			2.33 $_{ID. Music pmc}$			15.25 $_{ID. Leisure\_pmc}$			17.18 $_{ID. Leisure\_pmc}$		
	4.98 $_{ID. Music pmc}$						8.64 $_{ID. Music pmc}$			6.10 $_{ID. Music pmc}$		
ρ_{01}	-0.47 $_{ID.Time}$			0.01 $_{ID. Leisure\_pmc}$			-0.84			-0.29 $_{ID.Time}$		
	-0.22 $_{ID. Leisure\_pmc}$			0.05 $_{ID. Music pmc}$			0.29			-0.15 $_{ID. Leisure\_pmc}$		
	-0.10 $_{ID. Music pmc}$						-0.15			-0.24 $_{ID. Music pmc}$		
ICC	0.42			0.41			0.31			0.41		
N	711 $_{ID}$			711 $_{ID}$			711 $_{ID}$			711 $_{ID}$		
	7 $_{Day}$			7 $_{Day}$						7 $_{Day}$		
Observations	11981			11985			11987			11984		
Marginal R^2 / Conditional R^2	0.260 / 0.571			0.230 / 0.544			0.297 / 0.514			0.233 / 0.547		

Notes. Country=0(Austria)/1(Italy), DV=dependent variable, Gender=0(woman)/1(man), Leisure=0(no leisure activity)/1(leisure activity), Music=0(no previous music listening)/1(previous music listening), PHQ9=depressive symptom subscale of the Patient Health Questionnaire,[11] PSS=Perceived Stress Scale,[12] Time was centered on 10:00, Weekday=0(weekday)/1(weekend/holiday); GMC=grand mean centered, PM=person mean, pmc=person mean centered. Significant associations (p<.05) are marked in bold.

© 2023 Feneberg AC et al. *JAMA Network Open.*

eTable 4. Multilevel Models Predicting Dependent Variables by Music Listening, the Interaction Between Music Listening and Lagged DV, the Interaction Between Music Listening and Chronic Stress (PSS), and Covariates

	Momentary stress			Mood valence			Energetic arousal			Calmness		
Independent variable	*b*	*95%CI*	*p*	*b*	*95%CI*	*p*	*b*	*95%CI*	*p*	*b*	*95%CI*	*p*
(Intercept)	28.77	26.41 – 31.13	**<0.001**	65.14	63.39 – 66.89	**<0.001**	55.27	53.28 – 57.26	**<0.001**	61.50	59.66 – 63.33	**<0.001**
Music (pmc)	-0.83	-1.72 – 0.05	0.064	1.89	1.15 – 2.62	**<0.001**	2.55	1.65 – 3.45	**<0.001**	1.35	0.55 – 2.16	**0.001**
Lagged DV (pmc)	0.18	0.17 – 0.20	**<0.001**	0.24	0.23 – 0.26	**<0.001**	0.17	0.15 – 0.19	**<0.001**	0.21	0.19 – 0.23	**<0.001**
Time	-0.10	-0.42 – 0.22	0.542	-0.11	-0.18 – -0.05	**0.001**	0.81	0.48 – 1.13	**<0.001**	0.19	0.12 – 0.26	**<0.001**
Time^2	-0.02	-0.04 – -0.01	**0.012**				-0.21	-0.22 – -0.19	**<0.001**			
Leisure (pmc)	-5.50	-6.43 – -4.57	**<0.001**	3.24	2.49 – 3.99	**<0.001**	0.60	-0.19 – 1.39	0.135	4.07	3.30 – 4.85	**<0.001**
Weekday	-1.63	-2.35 – -0.90	**<0.001**	0.71	0.09 – 1.33	**0.025**	0.99	0.34 – 1.64	**0.003**	1.30	0.66 – 1.93	**<0.001**
Music (PM)	-0.57	-6.40 – 5.25	0.847	3.91	-0.97 – 8.79	0.117	1.88	-2.03 – 5.80	0.345	3.89	-1.22 – 9.00	0.136
PSS (GMC)	1.02	0.82 – 1.22	**<0.001**	-0.74	-0.91 – -0.57	**<0.001**	-0.22	-0.36 – -0.09	**0.001**	-0.93	-1.11 – -0.75	**<0.001**
Gender	1.45	-0.91 – 3.81	0.228	-2.05	-4.03 – -0.08	**0.042**	2.57	0.99 – 4.15	**0.001**	-2.35	-4.42 – -0.28	**0.026**
Age (GMC)	-0.11	-0.20 – -0.01	**0.032**	-0.02	-0.10 – 0.06	0.660	0.02	-0.05 – 0.08	0.596	0.05	-0.03 – 0.14	0.222
PHQ9 (GMC)	0.55	0.28 – 0.83	**<0.001**	-0.80	-1.03 – -0.57	**<0.001**	-0.30	-0.48 – -0.11	**0.002**	-0.58	-0.83 – -0.34	**<0.001**
Country	10.94	8.55 – 13.33	**<0.001**	-4.50	-6.50 – -2.50	**<0.001**	1.29	-0.31 – 2.89	0.114	-4.84	-6.93 – -2.75	**<0.001**
Music (pmc) * lagged DV (pmc)	-0.07	-0.11 – -0.02	**0.005**	-0.03	-0.08 – 0.02	0.223	-0.09	-0.13 – -0.04	**<0.001**	-0.03	-0.08 – 0.02	0.230
Music (pmc) * PSS (GMC)	-0.02	-0.14 – 0.10	0.738	0.12	0.02 – 0.22	**0.019**	0.15	0.03 – 0.27	**0.012**	0.06	-0.06 – 0.17	0.326
**Random Effects**												
σ^2	286.26			203.86			252.94			229.40		
τ_{00}	9.08 $_{ID:Day}$			9.70 $_{ID:Day}$			241.43 $_{ID}$			4.81 $_{ID:Day}$		
	224.47 $_{ID}$			125.66 $_{ID}$						138.98 $_{ID}$		
τ_{11}	0.24 $_{ID.Time}$			23.32 $_{ID. Leisure\_pmc}$			2.00 $_{ID.Time}$			22.22 $_{ID. Leisure\_pmc}$		
	45.00 $_{ID. Leisure\_pmc}$			1.73 $_{ID. Music pmc}$			15.52 $_{ID. Leisure\_pmc}$			8.83 $_{ID. Music pmc}$		
	5.02 $_{ID. Music pmc}$						7.47 $_{ID. Music pmc}$					
ρ_{01}	-0.47 $_{ID.Time}$			0.01 $_{ID. Leisure\_pmc}$			-0.84			-0.11 $_{ID. Leisure\_pmc}$		
	-0.22 $_{ID. Leisure\_pmc}$			0.05 $_{ID. Music pmc}$			0.28			-0.06 $_{ID. Music pmc}$		
	-0.11 $_{ID. Music pmc}$						-0.06					
ICC	0.42			0.41			0.31			0.39		
N	711 $_{ID}$			711 $_{ID}$			711 $_{ID}$			711 $_{ID}$		
	7 $_{Day}$			7 $_{Day}$						7 $_{Day}$		
Observations	11981			11985			11987			11984		
Marginal R^2 / Conditional R^2	0.260 / 0.571			0.230 / 0.544			0.297 / 0.514			0.235 / 0.536		

Notes. Country=0(Austria)/1(Italy), DV=dependent variable, Gender=0(woman)/1(man), Leisure=0(no leisure activity)/1(leisure activity), Music=0(no previous music listening)/1(previous music listening), PHQ9=depressive symptom subscale of the Patient Health Questionnaire,[11] PSS=Perceived Stress Scale,[12] Time was

centered on 10:00, Weekday=0(weekday)/1(weekend/holiday); GMC=grand mean centered, PM=person mean, pmc=person mean centered. Significant associations (p<.05) are marked in bold.

© 2023 Feneberg AC et al. *JAMA Network Open*.

eTable 5. Multilevel Models Predicting Dependent Variables by Music Characteristics and Covariates

	Momentary stress			Mood valence			Energetic arousal			Calmness		
Independent variable	*b*	*95%CI*	*p*	*b*	*95%CI*	*p*	*b*	*95%CI*	*p*	*b*	*95%CI*	*p*
(Intercept)	42.37	35.69 – 49.06	**<0.001**	53.21	47.86 – 58.57	**<0.001**	51.19	45.50 – 56.89	**<0.001**	49.93	44.32 – 55.55	**<0.001**
Musical valence (pmc)	-0.07	-0.12 – -0.02	**0.003**	0.08	0.04 – 0.12	**<0.001**	0.04	-0.01 – 0.09	0.107	0.08	0.04 – 0.12	**<0.001**
Musical arousal (pmc)	0.01	-0.03 – 0.04	0.718	0.01	-0.02 – 0.04	0.608	0.06	0.02 – 0.09	**0.002**	-0.00	-0.04 – 0.03	0.824
Time	-0.57	-1.27 – 0.14	0.115	-0.16	-0.31 – -0.00	**0.043**	0.02	-0.70 – 0.73	0.962	0.29	0.14 – 0.45	**<0.001**
Time²	0.00	-0.04 – 0.04	0.892				-0.16	-0.20 – -0.13	**<0.001**			
Leisure (pmc)	-5.14	-6.85 – -3.43	**<0.001**	3.31	1.87 – 4.76	**<0.001**	0.40	-1.23 – 2.03	0.630	3.40	1.87 – 4.93	**<0.001**
Weekday	-1.37	-2.96 – 0.22	0.092	0.93	-0.40 – 2.27	0.171	2.06	0.59 – 3.52	**0.006**	1.36	-0.06 – 2.77	0.060
Lagged DV (pmc)	0.14	0.10 – 0.18	**<0.001**	0.24	0.21 – 0.28	**<0.001**	0.10	0.06 – 0.14	**<0.001**	0.17	0.13 – 0.21	**<0.001**
Musical valence (PM)	-0.17	-0.27 – -0.08	**<0.001**	0.20	0.12 – 0.28	**<0.001**	0.11	0.04 – 0.19	**0.004**	0.18	0.10 – 0.27	**<0.001**
Musical arousal (PM)	-0.02	-0.10 – 0.07	0.718	0.01	-0.06 – 0.09	0.712	0.03	-0.04 – 0.10	0.465	0.01	-0.07 – 0.09	0.849
PSS (GMC)	0.93	0.69 – 1.18	**<0.001**	-0.68	-0.89 – -0.47	**<0.001**	-0.09	-0.29 – 0.10	0.348	-0.86	-1.08 – -0.64	**<0.001**
Gender	0.61	-2.26 – 3.48	0.679	-0.59	-3.04 – 1.86	0.636	3.41	1.14 – 5.67	**0.003**	-1.71	-4.28 – 0.85	0.191
Age (GMC)	-0.17	-0.29 – -0.05	**0.005**	-0.01	-0.11 – 0.10	0.903	-0.01	-0.10 – 0.09	0.908	0.07	-0.04 – 0.18	0.206
PHQ9 (GMC)	0.48	0.14 – 0.82	**0.005**	-0.59	-0.87 – -0.30	**<0.001**	-0.30	-0.57 – -0.04	**0.026**	-0.45	-0.75 – -0.15	**0.004**
Country	8.86	5.98 – 11.73	**<0.001**	-3.47	-5.93 – -1.02	**0.005**	1.94	-0.34 – 4.21	0.095	-3.35	-5.92 – -0.78	**0.011**
**Random Effects**												
σ^2	285.77			211.40			268.70			233.86		
τ_{00}	19.51 $_{ID:Day}$			8.02 $_{ID:Day}$			182.65 $_{ID}$			11.40 $_{ID:Day}$		
	146.25 $_{ID}$			107.89 $_{ID}$						117.27 $_{ID}$		
τ_{11}							1.36 $_{ID.Time}$					
ρ_{01}							-0.79 $_{ID}$					
ICC	0.37			0.35			0.26			0.35		
N	599 $_{ID}$			599 $_{ID}$			599 $_{ID}$			599 $_{ID}$		
	7 $_{Day}$			7 $_{Day}$						7 $_{Day}$		
Observations	2664			2664			2665			2664		
Marginal R^2 / Conditional R^2	0.250 / 0.525			0.231 / 0.503			0.338 / 0.509			0.223 / 0.499		

Notes. Country=0(Austria)/1(Italy), DV=dependent variable, Gender=0(woman)/1(man), Leisure=0(no leisure activity)/1(leisure activity), Musical valence=0(sad) to 100 (happy), Musical arousal=0(calming) to 100 (energizing), PHQ9=depressive symptom subscale of the Patient Health Questionnaire,[11] PSS=Perceived Stress Scale,[12] Time was centered on 10:00, Weekday=0(weekday)/1(weekend/holiday); GMC=grand mean centered, PM=person mean, pmc=person mean centered. Significant associations (p<.05) are marked in bold.

© 2023 Feneberg AC et al. *JAMA Network Open.*

eTable 6. Multilevel Models Predicting Dependent Variables by Reasons for Music Listening and Covariates

	**Momentary stress**			**Mood valence**			**Energetic arousal**			**Calmness**		
Independent variable	*b*	*95%CI*	*p*	*b*	*95%CI*	*p*	*b*	*95%CI*	*p*	*b*	*95%CI*	*p*
(Intercept)	31.38	26.25 – 36.50	**<0.001**	63.37	59.40 – 67.33	**<0.001**	58.71	54.16 – 63.26	**<0.001**	56.42	52.31 – 60.54	**<0.001**
Relaxation (pmc)	1.91	-0.93 – 4.74	0.187	0.08	-2.33 – 2.49	0.948	-0.87	-3.59 – 1.86	0.533	-0.50	-3.05 – 2.04	0.699
Activation (pmc)	1.34	-1.46 – 4.14	0.349	0.82	-1.56 – 3.21	0.499	1.23	-1.47 – 3.94	0.371	0.76	-1.76 – 3.27	0.555
Distraction (pmc)	4.16	1.04 – 7.29	**0.009**	-1.82	-4.48 – 0.84	0.179	0.90	-2.11 – 3.91	0.558	-1.36	-4.16 – 1.45	0.343
Reducing boredom (pmc)	1.21	-2.14 – 4.57	0.479	0.17	-2.69 – 3.03	0.908	1.39	-1.85 – 4.62	0.401	0.70	-2.32 – 3.72	0.649
Time	-0.59	-1.29 – 0.11	0.100	-0.16	-0.31 – -0.01	**0.043**	0.09	-0.62 – 0.81	0.802	0.30	0.14 – 0.46	**<0.001**
Time^2	0.00	-0.04 – 0.04	0.827				-0.17	-0.21 – -0.13	**<0.001**			
Leisure (pmc)	-5.11	-6.83 – -3.40	**<0.001**	3.31	1.85 – 4.76	**<0.001**	0.47	-1.16 – 2.11	0.570	3.42	1.89 – 4.96	**<0.001**
Weekday	-1.39	-3.00 – 0.22	0.091	0.95	-0.40 – 2.30	0.167	2.06	0.59 – 3.53	**0.006**	1.37	-0.06 – 2.80	0.060
Lagged DV (pmc)	0.13	0.09 – 0.17	**<0.001**	0.25	0.21 – 0.29	**<0.001**	0.11	0.07 – 0.15	**<0.001**	0.17	0.13 – 0.21	**<0.001**
Relaxation (PM)	-1.10	-7.00 – 4.80	0.715	5.73	0.64 – 10.81	**0.027**	-1.71	-6.42 – 2.99	0.476	8.54	3.28 – 13.80	**0.001**
Activation (PM)	-4.09	-9.66 – 1.47	0.149	7.56	2.77 – 12.35	**0.002**	4.53	0.08 – 8.98	**0.046**	9.27	4.31 – 14.23	**<0.001**
Distraction (PM)	-1.93	-9.01 – 5.14	0.592	1.32	-4.77 – 7.42	0.670	-2.94	-8.57 – 2.70	0.307	3.89	-2.42 – 10.19	0.227
Reducing boredom (PM)	0.16	-7.87 – 8.19	0.968	3.57	-3.35 – 10.49	0.312	4.83	-1.54 – 11.19	0.137	6.35	-0.80 – 13.51	0.082
PSS (GMC)	0.95	0.70 – 1.19	**<0.001**	-0.69	-0.90 – -0.47	**<0.001**	-0.09	-0.28 – 0.11	0.376	-0.87	-1.09 – -0.65	**<0.001**
Gender	1.67	-1.22 – 4.56	0.256	-1.68	-4.17 – 0.81	0.185	3.31	1.05 – 5.58	**0.004**	-2.76	-5.33 – -0.18	**0.036**
Age (GMC)	-0.17	-0.29 – -0.04	**0.008**	0.01	-0.10 – 0.12	0.855	0.02	-0.08 – 0.12	0.668	0.09	-0.02 – 0.20	0.102
PHQ9 (GMC)	0.56	0.21 – 0.90	**0.002**	-0.66	-0.96 – -0.36	**<0.001**	-0.33	-0.60 – -0.06	**0.016**	-0.53	-0.84 – -0.23	**0.001**
Country	9.46	6.50 – 12.42	**<0.001**	-4.00	-6.55 – -1.45	**0.002**	2.13	-0.19 – 4.45	0.072	-3.75	-6.39 – -1.11	**0.005**
**Random Effects**												
σ^2	281.61			210.89			271.00			232.63		
τ_{00}	25.55 $_{\text{ID:Day}}$			10.59 $_{\text{ID:Day}}$			198.20 $_{\text{ID}}$			14.58 $_{\text{ID:Day}}$		
	151.65 $_{\text{ID}}$			115.25 $_{\text{ID}}$						120.05 $_{\text{ID}}$		
τ_{11}							1.34 $_{\text{ID.Time}}$					
ρ_{01}							-0.82 $_{\text{ID}}$					
ICC	0.39			0.37			0.26			0.37		
N	599 $_{\text{ID}}$			599 $_{\text{ID}}$			599 $_{\text{ID}}$			599 $_{\text{ID}}$		
	7 $_{\text{Day}}$			7 $_{\text{Day}}$						7 $_{\text{Day}}$		
Observations	2664			2664			2665			2664		
Marginal R^2 / Conditional R^2	0.236 / 0.531			0.208 / 0.504			0.329 / 0.503			0.212 / 0.501		

Notes. Country=0(Austria)/1(Italy), DV=dependent variable, Gender=0(woman)/1(man), Leisure=0(no leisure activity)/1(leisure activity), Relaxation/Activation/Distraction/Reducing boredom=0(not selected)/1(selected), PHQ9=depressive symptom subscale of the Patient Health Questionnaire,[11] PSS=Perceived Stress Scale,[12] Time was centered on 10:00, Weekday=0(weekday)/1(weekend/holiday); GMC=grand mean centered, PM=person mean, pmc=person mean centered. Significant associations (p<.05) are marked in bold.

© 2023 Feneberg AC et al. *JAMA Network Open.*

eReferences.

1. Feneberg AC, Forbes PAG, Piperno G, et al. Diurnal dynamics of stress and mood during COVID-19 lockdown: a large multinational ecological momentary assessment study. *Proc. Biol. Sci.* 2022;289(1975):20212480. doi: 10.1098/rspb.2021.2480.
2. Forbes PAG, Pronizius E, Feneberg AC, et al. The effects of social interactions on momentary stress and mood during COVID-19 lockdowns. *Br J Health Psychol*. 2022. doi: 10.1111/bjhp.12626.
3. Stijovic A, Forbes P, Tomova L, et al. *Homeostatic regulation of energetic arousal during acute social isolation-converging evidence from the lab and the field*. PsyArXiv. 2022. doi: 10.31234/osf.io/3z74g.
4. Trull TJ, Ebner-Priemer UW. Ambulatory assessment in psychopathology research: A review of recommended reporting guidelines and current practices. *Journal of abnormal psychology*. 2020;129(1):56-63. doi: 10.1037/abn0000473.
5. Mewes R, Feneberg AC, Doerr JM, Nater UM. Psychobiological mechanisms in somatic symptom disorder and depressive disorders - An ecological momentary assessment approach. *Psychosomatic medicine*. 2022;84(1):86–96. doi: 10.1097/PSY.0000000000001006.
6. Doerr JM, Ditzen B, Strahler J, et al. Reciprocal relationship between acute stress and acute fatigue in everyday life in a sample of university students. *Biological psychology*. 2015;110:42-49. doi: 10.1016/j.biopsycho.2015.06.009.
7. Enders CK, Tofighi D. Centering predictor variables in cross-sectional multilevel models: a new look at an old issue. *Psychological Methods*. 2007;12(2):121-138. doi: 10.1037/1082-989X.12.2.121.
8. Baird R, Maxwell SE. Performance of time-varying predictors in multilevel models under an assumption of fixed or random effects. *Psychological Methods*. 2016;21(2):175-188. doi: 10.1037/met0000070.
9. Matuschek H, Kliegl R, Vasishth S, Baayen H, Bates D. Balancing Type I error and power in linear mixed models. *J. Mem. Lang.* 2017;94:305-315. doi: 10.1016/j.jml.2017.01.001.
10. Nater UM, Krebs M, Ehlert U. Sensation Seeking, Music Preference, and Psychophysiological Reactivity to Music. *Musicae Scientiae*. 2005;9(2):239-254. doi: 10.1177/102986490500900205.
11. Löwe B, Spitzer RL, Zipfel S, Herzog W. *Gesundheitsfragebogen für Patienten (PHQ D). Komplettversion und Kurzform*. [Testmappe mit Manual, Fragebögen, Schablonen]. Karlsruhe: Pfizer; 2002.
12. Cohen S, Kamarck T, Mermelstein R. A global measure of perceived stress. *Journal of health and social behavior*. 1983;24(4):385-396.

© 2023 Feneberg AC et al. *JAMA Network Open.*
