# Supplementary material for: Perceptions of Stress and Mood Associated With Listening to Music in Daily Life During the COVID-19 Lockdown
Source: JAMA Netw Open. 2023 Jan 10;6(1):e2250382. doi: 10.1001/jamanetworkopen.2022.50382 (PMC9857599; doi:10.1001/jamanetworkopen.2022.50382)
Supplement: Supplement 2. — Data Sharing Statement [file jamanetwopen-e2250382-s002.pdf]

Data Sharing Statement

Feneberg. Perceptions of Stress and Mood Associated With Listening to Music in Daily Life During the COVID-19 Lockdown. *JAMA Netw Open*. Published January 10, 2023. doi:10.1001/jamanetworkopen.2022.50382

Data
Data available: Yes
Data types: Deidentified participant data
How to access data: https://osf.io/q3uz9/
When available: beginning date: 11-06-2022

Supporting Documents
Document types: Statistical/analytic code
How to access documents: https://osf.io/q3uz9/
When available: beginning date: 11-06-2022

Additional Information
Who can access the data: anyone
Types of analyses: for any purpose
Mechanisms of data availability: without investigator support
